# Motor Control and Sensory Feedback Enhance Prosthesis Embodiment and Reduce Phantom Pain After Long-Term Hand Amputation

**DOI:** 10.3389/fnhum.2018.00352

**Published:** 2018-09-21

**Authors:** David M. Page, Jacob A. George, David T. Kluger, Christopher Duncan, Suzanne Wendelken, Tyler Davis, Douglas T. Hutchinson, Gregory A. Clark

**Affiliations:** ^1^Department of Bioengineering, University of Utah, Salt Lake City, UT, United States; ^2^Division of Physical Medicine and Rehabilitation, University of Utah, Salt Lake City, UT, United States; ^3^Department of Neurosurgery, University of Utah, Salt Lake City, UT, United States; ^4^Department of Orthopaedics, University of Utah, Salt Lake City, UT, United States

**Keywords:** prosthesis embodiment, phantom pain, neural prosthetics, neuroprostheses, sensory feedback, prosthesis ownership, hand ownership

## Abstract

We quantified prosthesis embodiment and phantom pain reduction associated with motor control and sensory feedback from a prosthetic hand in one human with a long-term transradial amputation. Microelectrode arrays were implanted in the residual median and ulnar arm nerves and intramuscular electromyography recording leads were implanted in residual limb muscles to enable sensory feedback and motor control. Objective measures (proprioceptive drift) and subjective measures (survey answers) were used to assess prosthesis embodiment. For both measures, there was a significant level of embodiment of the physical prosthetic limb after open-loop motor control of the prosthesis (i.e., without sensory feedback), open-loop sensation from the prosthesis (i.e., without motor control), and closed-loop control of the prosthesis (i.e., motor control with sensory feedback). There was also a statistically significant reduction in reported phantom pain after experimental sessions that included open-loop nerve microstimulation, open-loop prosthesis motor control, or closed-loop prosthesis motor control. The closed-loop condition provided no additional significant improvements in phantom pain reduction or prosthesis embodiment relative to the open-loop sensory condition or the open-loop motor condition. This study represents the first long-term (14-month), systematic report of phantom pain reduction and prosthesis embodiment in a human amputee across a variety of prosthesis use cases.

## Introduction

The emotional, psychological, and functional effects of upper limb amputation can be devastating. Many amputees undergo a period of mourning, a chronic struggle with depression, and endurance of life-long phantom pain (Marshall et al., 1992; Desmond and MacLachlan, 2006; Bhuvaneswar et al., 2007; Ziegler-Graham et al., 2008; Hanley et al., 2009), in addition to practical difficulties associated with activities of daily living (ADL) and potential loss of employment. These challenges often result in long-term use of antidepressants and narcotics and ongoing medical costs associated with anxiety and other psychological struggles (van der Schans et al., 2002; Jensen et al., 2007). We hypothesize that engagement with a motorized, sensorized prosthetic hand will enhance prosthesis embodiment—i.e., meaningful integration of the prosthesis into one’s body image—and phantom pain reduction. Such consequences, together with sophisticated functional prosthesis use, may in turn improve many of these aspects of life for amputees, and may result in substantial cost savings to healthcare organizations and payment agencies.

The current standard-of-care after upper limb amputation includes four basic options: (1) use of a body-powered hook; (2) use of a myoelectric hook or hand prosthesis; (3) use of a non-functional cosmetic prosthesis; or (4) use of the residual limb (i.e., no prosthesis) (Biddiss and Chau, 2007). Most body-powered hooks, myoelectric prostheses, and cosmetic prostheses do not currently provide sensory feedback directly, and motor control of these prostheses is often limited to only 1–3 degrees of freedom (DOF) that typically are not controllable simultaneously. Many amputees prefer to use their residual limb instead of a prosthesis, which has been proposed to be due in part to the presence of sensory feedback (Raichle et al., 2008). Furthermore, for commercially-available prostheses, the residual limb does not provide the sophisticated multi-DOF motor control provided by an intact hand.

Peripheral nerve and muscle interfaces offer an exciting opportunity to provide subjects with improved prosthesis control and sensory feedback. Many different peripheral-nerve interfaces have been used, including transverse intrafascicular multichannel electrodes (TIMEs) (Raspopovic et al., 2014), flat-interface nerve electrodes (FINEs) (Tan et al., 2014), and longitudinal intrafascicular electrodes (LIFEs) (Dhillon et al., 2004, 2005; Dhillon and Horch, 2005; Horch et al., 2011; Graczyk et al., 2016). Regular use of commercially available myoelectric prosthetics can reduce phantom limb pain (Lotze et al., 1999), and improved prosthesis control and sensory feedback via peripheral nerve interfaces may further reduce phantom pain. Synchronized visual and motor feedback (Ramachandran and Rogers-Ramachandran, 1996) or visual and sensory feedback (Schmalzl et al., 2013) can alleviate phantom pain, and initial evidence suggests that prosthesis sensory feedback can also reduce phantom pain (Dietrich et al., 2012; Tan et al., 2014). However, there has been no systematic study of phantom pain reduction when using these peripheral nerve interfaces for sensory feedback or for motor control.

Basic motor control has been provided to amputees using implanted myoelectric sensors (IMES) and fine wire muscle electrodes, but outcome measures have focused largely on functional performance (Baker et al., 2008; Weir et al., 2009; Cipriani et al., 2014; Smith et al., 2014), with few reports on psychological and emotional impact metrics such as prosthesis embodiment and/or pain reduction in response to prosthesis motor control (Rosén et al., 2009; Ortiz-Catalan et al., 2014; Souza et al., 2014). Implants have also been placed in the central nervous system for the purpose of restoring prostheses motor control and sensory feedback (Kim et al., 2011; O’Doherty et al., 2011; Hochberg et al., 2012; Tabot et al., 2013; Flesher et al., 2016); however, most amputees are unwilling to undergo brain surgery (Engdahl et al., 2015, 2017). Targeted muscle reinnervation has also been used to restore basic sensory and motor feedback to human amputees (Kuiken et al., 2004, 2007; O’Shaughnessy et al., 2008; Hebert et al., 2016), and prosthesis embodiment was enhanced for two lower-limb amputees using sensory feedback alone (Marasco et al., 2011). Cortical stimulation has been shown to enhance prosthesis embodiment in two intact human subjects (Collins et al., 2017), but this approach was also limited to open-loop sensory-feedback trials in which sensory feedback was provided to only a single hand location.

To date, enhanced prosthesis embodiment due to closed-loop control of the prosthesis has been demonstrated only with two upper-limb amputees implanted with FINE electrodes (Schiefer et al., 2016). This study did not include quantitation of the subjects’ perceived phantom hand location, nor did it quantify embodiment relatively across multiple prosthesis use cases. Other reports of upper-limb amputees’ embodiment of closed-loop prostheses utilized referred sensations from the residual limb instead of direct neural stimulation (Rosén et al., 2009).

We have previously reported functional performance improvements due to closed-loop control using Utah Slanted Electrode Arrays (USEAs) (Clark et al., 2014; Davis et al., 2016; Wendelken et al., 2017). As a complement to the functional outcomes, we here report on the psychological impact of advanced prosthesis control and sensation. We report embodiment of a physical prosthesis during closed-loop, multiple-DOF prosthesis control with multi-channel sensory feedback from different hand locations in a single human amputee. We also report embodiment due to open-loop motor control, as well as embodiment due to multi-sensor open-loop touch-feedback from the prosthetic hand. This is in contrast to past embodiment studies which used only open-loop sensory feedback (Marasco et al., 2011; Collins et al., 2017), or closed-loop sensory feedback through referred sensations (Rosén et al., 2009).

This work represents a case study of our first use of closed-loop physical prosthesis control with USEA-evoked sensory feedback in one human amputee. Additionally, to our knowledge, this is the first report using perceptual phantom hand location as an objective measurement of prosthesis embodiment for closed-loop controlled prostheses where feedback is provided via peripheral nerve microstimulation. This objective metric for embodiment has been used primarily in previous studies with intact subjects (Botvinick and Cohen, 1998; Tsakiris et al., 2006; Kalckert and Ehrsson, 2012; Caspar et al., 2015; Jenkinson and Preston, 2015; Romano et al., 2015), once with an amputee receiving sensory feedback from referred sensations (Rosén et al., 2009), and only once with intact individuals receiving sensory feedback from cortical stimulation (Collins et al., 2017). This metric has never been used to assess embodiment due to closed-loop prosthesis use with amputees receiving sensory feedback from peripheral nerve microstimulation. We also provide a 14-month report of phantom pain reduction for the subject due to participation in experiments including USEA microstimulation, open-loop prosthesis control, and closed-loop prosthesis control.

## Materials and Methods

### Study Volunteer

We implanted USEAs and electromyographic recording leads (iEMGs) in one transradial amputee. A similar approach was used with other amputees in prior publications from this group (Clark et al., 2014; Davis et al., 2016; Dantas et al., 2017, 2018; Nieveen et al., 2017; Wendelken et al., 2017; Zhang et al., 2017; George et al., 2018). The subject was recruited by a physician and evaluated by a psychologist prior to participating in the study. The subject was a 57-year-old, left-hand-dominant male, whose left foot and left forearm had been amputated 13 years prior, after an electrocution injury. His unilateral, left-arm amputation was midway along the forearm, leaving many extrinsic hand muscles intact. The subject indicated that he generally preferred to use his residual arm instead of a prosthesis, although he occasionally used a body-powered hook for work around his home and a basic rubber-handed myoelectric prosthesis for cosmetic purposes at social gatherings.

Several years prior to these experiments, the subject received experimental nerve-interface implants on two occasions in his amputated left arm residual nerves. These prior experiments involved implantation of intraneural electrodes for a duration of up to 2 weeks each, with experiments including electrode recording and stimulation for motor control and sensory feedback from a simple physical prosthesis. We do not anticipate that these prior experiments substantially impacted the current results, other than perhaps increasing the subject’s learning speed for some of the experimental tasks, although some residual nerve damage or other consequences from prior implants and experiments cannot be entirely ruled out.

Preimplant training included mimicking motor hand movements displayed on a video (Clark et al., 2014; Davis et al., 2016; Dantas et al., 2017, 2018; Wendelken et al., 2017; Zhang et al., 2017; George et al., 2018), as well as tactile stimulation training on the skin of his residual limb and his intact hand using a mechanical vibrometer. The subject routinely used gabapentin (800 mg, typically 2–4 times per day), ibuprofen (800 mg, typically 0–4 times per day), amitriptyline (25 mg, typically 0–1 times per day) and tramadol (1000 mg, typically 0–2 times per day) both prior to and during the implant period. The subject’s medication use was monitored and documented throughout the study in order to have an unbiased assessment of phantom pain as a result from participating in experimental sessions. The most recent medications prior to the start of each experimental session consistently included tramadol (9–12 h prior), amitriptyline (9 h prior), and gabapentin (9–12 h prior and 1–4 h prior). Informed consent and experimental protocols were carried out in accordance with the University of Utah Institutional Review Board and the Department of the Navy Human Research Protection Program.

### Devices

Two Utah Slanted Electrode Arrays (USEAs; Blackrock Microsystems, Salt Lake City, UT, United States) were implanted in the subject’s residual limb proximal to the elbow: one in the median nerve, and the other in the ulnar nerve. USEAs are silicon microelectrode arrays with 100 electrode shafts on each USEA arranged in a 10 × 10 grid on a 4-mm × 4-mm base. Electrode shafts are spaced 400 μm apart, with lengths of shafts varying along a single dimension from ∼0.75 to 1.5 mm (Branner et al., 2001). The USEAs used for these experiments had iridium oxide tips and parylene-C insulation. Four looped platinum wires were also implanted—two served as electrical ground and stimulation return, and two served as reference wires for recording. Four electrodes from the longest row of electrode shafts on the USEA were also sometimes used as an on-array electrical reference for recordings (Clark et al., 2011, 2014). The ground and reference wires, as well as the electrodes on the USEAs, were wired-bonded to external connectors with helically-wound, silicone-potted wires and traveled through a percutaneous incision to allow connection via active or passive Gator Connector Cables (Ripple LLC, Salt Lake City, UT, United States).

Eight intramuscular electromyographic recording leads (iEMGs; Ripple LLC, Salt Lake City, UT, United States) were implanted in the residual arm muscles, with attempted targeting of each lead to different lower-arm extensor or flexor muscles. Each of the eight leads contained four electrical contacts, totaling 32 recording channels. A separate iEMG lead was implanted proximal and posterior to the elbow to provide contacts for an electrical reference and ground. The implanted EMG electrodes were also wired via a percutaneous incision to an external Gator Connector Board (Ripple LLC, Salt Lake City, UT, United States).

### Surgical Implant

**FIGURE 1:**
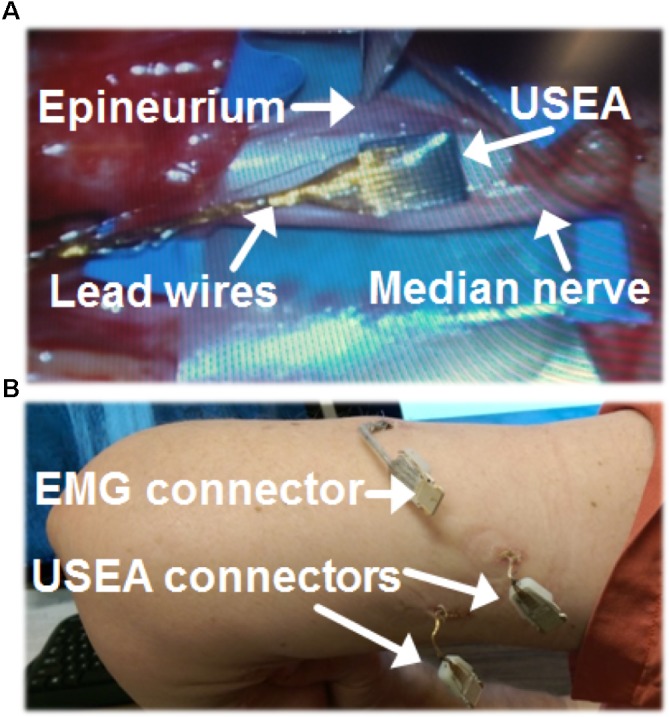
Surgical methods for Utah Slanted Electrode Arrays (USEAs) and intramuscular electromyographic implants (iEMG). **(A)** The epineurium was separated prior to implantation of a USEA in the median nerve. A USEA was also implanted in the ulnar nerve (not shown). **(B)** USEA and iEMG lead wires were connected to the contact pads of external connector boards via percutaneous incisions. Hardware was attached to these connector boards during experiments to enable stimulation and recording via the USEA and iEMG implants.

Starting the day before the implant surgery, the subject was given an oral prophylactic antibiotic (100 mg minocycline, 7 days, twice per day), which has been reported to improve neuronal recording quality in rats (Rennaker et al., 2007). Under general anesthesia, the USEAs were placed in median and ulnar nerves in the upper arm, several centimeters proximal to the medial epicondyle. The iEMGs were implanted midway along the forearm. After dissection of the epineurium, USEAs were implanted using a pneumatic inserter tool (Rousche and Normann, 1992). The epineurium was sutured around each USEA and its ground and reference wires (**Figure 1A**). A collagen wrap (AxoGen Inc., Alachua, FL, United States) was placed around the median-nerve USEA and secured with vascular clips. Collagen wrap was not placed around the USEA in the ulnar nerve, due to limited tourniquet time and the presence of scar tissue near the desired implant site from a previous intrafascicular nerve stimulation study. A 0.1 mg/kg dose of dexamethasone was administered after tourniquet removal, which has been reported to reduce the foreign body response and improve neural recordings (Spataro et al., 2005; Zhong and Bellamkonda, 2007).

The percutaneous wire sites (**Figure 1B**) were dressed using an antibiotic wound patch (Biopatch, Ethicon US LLC, Somerville, NJ, United States) at least every 10 days. The implants remained intact in the subject for 63 weeks and one local infection at the iEMG implant site early in the implant period was successfully resolved with oral antibiotics (keflex and bactrim) administered for 2–3 weeks. The subject participated in 2–3 h experimental sessions typically 1–4 days per week. Experimental sessions included motor-decode training and testing (via iEMG and/or USEA recordings), sensory-encode training and testing (via USEA stimulation), and closed-loop control assessments (via simultaneous recording from USEAs and/or iEMGs and stimulation via USEAs) as well as impedance testing of the USEAs and iEMGs at the beginning and end of each session. At the end of the 63 weeks, the USEAs and iEMGs were surgically removed from the study as the result of prior mutual agreements between the volunteer subject and the experimenters regarding study duration.

### Recording/Decode

Neural and electromyography recording were collected using the 512-channel Grapevine System (Ripple LLC, Salt Lake City, UT, United States). Although neural recordings and iEMG recordings both served as motor-decode features, iEMG recordings were used exclusively for the vast majority of experimental sessions. In addition, iEMG recordings were used exclusively for all embodiment experiments. The 32 single-ended EMG signals were acquired at 1 kHz. The raw EMG signals were then filtered with a 6th-order high-pass Butterworth (cutoff of 15 Hz), a 2nd-order low-pass Butterworth filter (cutoff of 375 Hz), and 60, 120, and 180 Hz notch filters. Differential EMG signals for all 496 possible combinations of channels were then calculated and the mean absolute value was calculated at 30 Hz. The resulting mean absolute value was then smoothed using an overlapping 300-ms rectangular window. The resulting feature dataset consisted of 528 channels (32 single-ended electrodes + 496 differential pairs) sampled at 30 Hz. Neural recordings, when used, contributed an additional 196 features and were processed as described in Wendelken et al. (2017).

Recordings from iEMGs and USEAs were collected while the subject mimicked a set of preprogrammed virtual hand training movements with his phantom hand, which included individuated movements of different DOF (e.g., flexions/extensions of each digit, wrist flexion/extension, wrist pronation/supination, thumb abduction/adduction). Training sets included 5–10 trials for each training movement.

Outputs of selected channels from the feature dataset, as well as the instructed positions of each DOF from the training, were used to fit the parameters of a Kalman filter. The baseline firing-rate activity for each channel was subtracted from the overall firing rate prior to training and testing of the Kalman filter. Selection of channels for input into the Kalman filter was performed by a stepwise Gram-Schmidt electrode-selection algorithm (Nieveen et al., 2017). The output of the Kalman filter was modified using thresholds and gains, as also used previously (George et al., 2018). The effect of these modifications can be written as

$$Modified\;Output\text{=}\{\begin{array}{rr} \frac{\left(Output\;\cdot \;Gain\;-\;Threshold\right)}{1-Threshold}, & \text{​​}Output\geq Threshold\\ 0, & Output<Threshold \end{array}\}$$

The default values of the thresholds and gains were initially set to 0.2 and 1.0, respectively, although they were often subsequently tuned on an individual DOF basis to provide optimal control. This modified Kalman filter enabled the subject to proportionally control movements of either a virtual prosthetic hand or a physical prosthetic hand in real time. The modified Kalman filter output was either used directly for real-time position or velocity control.

### Stimulation/Encode

Electrical stimulation was delivered via single or multiple USEA electrodes using the Grapevine System with Micro2+Stim front ends. All stimulation was delivered as biphasic, cathodic-first pulses, with 200–320-μs phase durations, and a 100-μs interphase duration. The stimulation frequency varied between 10–500 Hz, and stimulation amplitudes were in the range of 1–100 μA.

### Closed-Loop Control

Closed-loop control (i.e., motor control with USEA-coupled sensory feedback) was provided to the subject after performing motor-decode and sensory-encode training. Sensory-encode training consisted of identifying electrodes that evoked percepts that could be associated with sensor locations on the virtual or physical hand. Typically, the assigned electrodes evoked sensory percepts with projected fields that matched the location of sensors on the hand. The frequency of stimulation on an assigned electrode was mapped to be roughly proportional to the indentation force of the sensor in real time, although stochastic variability was added to the stimulation frequency to produce a firing pattern more reminiscent of biologically-generated firing patterns. Closed-loop control sessions included performance of tasks with either the virtual prosthetic hand or the physical prosthetic hand. During virtual prosthesis use, the position of the residual limb relative to the subject’s body was tracked and mapped to the virtual hand using a motion tracking system (OptiTrack, Corvallis, OR, United States).

### Physical and Virtual Prosthesis

During embodiment experiments, the subject used one of two physical prostheses. During the first 9 months of the study, the subject used a custom 3D-printed Ada Hand (Open Bionics, Bristol, United Kingdom). For the final 5 months of the study, the subject used the newly released, more advanced DEKA LUKE Arm (DEKA, Manchester, NH, United States).

**FIGURE 2:**
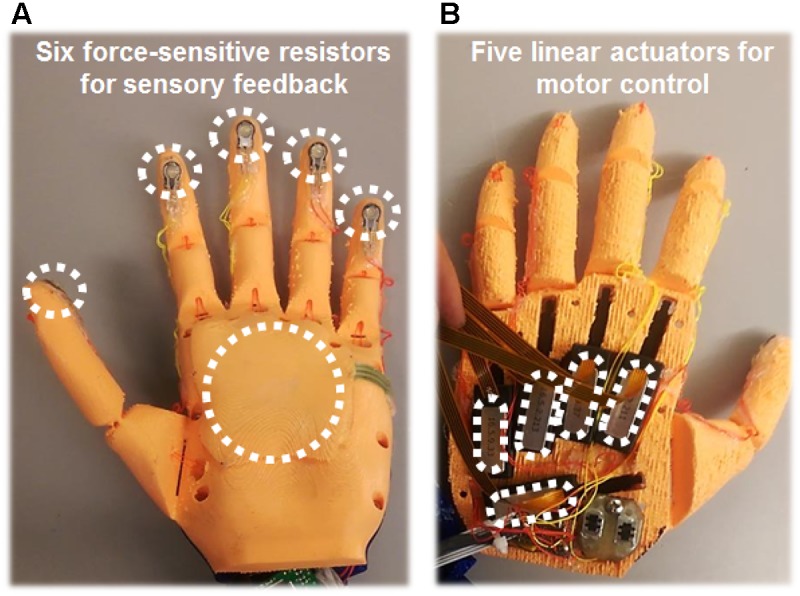
3D-printed physical prosthetic hand used for embodiment experiments. **(A)** Six force-sensitive resistors were fixed to the prosthetic hand: one sensor on each digit tip, and a larger sensor on the palm. Activation of these sensors produced increased USEA stimulation and associated sensations on the phantom hand. Typically, the USEA electrode(s) assigned to each sensor evoked a sensory percept that corresponded to the same hand region as the sensor. Due to hardware limitations, a maximum of four prosthesis sensors were used simultaneously. **(B)** On the back of the hand, a linear actuator was attached to the tip of each digit of the prosthetic hand via a plastic cable that acted as an artificial tendon. Motor control signals were generated by decoding recordings from 32 electromyography contacts (eight leads, with four contacts each) implanted in the forearm muscles of the residual limb. During most embodiment experiments, the subject was able to control flexion and relaxation of all five digits of the prosthetic hand independently.

The first prosthesis, the custom Ada Hand (**Figure 2**), was instrumented with PQ-12 linear actuators on each digit (Firgelli Technologies, Victoria, BC, Canada) and 0.5-cm-diameter circular, flat, force-sensitive resistors on each digit tip and a 4-cm × 4-cm, square, flat, force-sensitive resistor on the palm (Interlink Electronics, Westlake Village, CA, United States). The physical hand was interfaced with custom software and the Ripple Grapevine System via an Almond board (Open Bionics, Bristol, United Kingdom) that allowed real-time feedback control via all five motors and via four of the six sensors during use. The physical prosthesis was 3D-printed with peach-colored filament, and a translucent, nude-Caucasian-tinted surgeon’s glove was placed over it to cover the electronics and sensors, approximating the subject’s skin tone.

The second prosthesis, the DEKA LUKE Arm, in its transradial configuration, had six DOFs (rotation of the wrist, abduction/adduction of the thumb, and flexion/extension of the wrist, thumb, and index finger, and coupled flexion/extension of the remaining three digits). The DEKA LUKE Arm had six position sensors (one for each DOF) and 13 contact sensors (two on the palm, one on the lateral edge of the palm, one on the back of the hand, four on the distal portion of the thumb, one on the lateral portion of the index finger, and one on each of the three remaining digits). A semi-translucent rubber glove covered the DEKA LUKE Arm, and served in place of the nude-Caucasian-tinted surgeon’s glove.

The virtual prosthesis was simulated and visualized by either MSMS (Davoodi et al., 2007) or the MuJoCo virtual reality environment (Roboti LLC, Redmond, WA, United States). The MSMS hand was a virtual Modular Prosthetic Limb (Johns Hopkins Applied Physics Lab, Baltimore, MD, United States) used only for open-loop motor-decode training, and the MuJoCo hand was a virtual model of the LUKE Arm used for both open-loop and closed-loop-control tasks using integrated virtual sensors. Subjective pain scores were measured before and after experimental sessions using both physical and virtual prostheses.

### Embodiment Experiments

We assessed the level of embodiment of the physical prosthetic hands via two metrics: (1) comparison of the subject’s perceived phantom-hand position from before versus after an embodiment training period; and (2) collection of survey responses related to prosthesis embodiment. Both metrics have been shown to quantify embodiment (i.e., ownership of a body part) without addressing agency (i.e., the feeling of control over bodily actions) (Kalckert and Ehrsson, 2012). Quantification of agency is not reported in this study.

A total of ten experimental sets were performed, seven with the Open Bionics prosthesis and three with the DEKA prosthesis. For statistical analysis, we aggregated the data for the two prostheses into a single dataset. A direct comparison of the level of embodiment of the two different prostheses was beyond the intended scope of the study and would be confounded by temporal factors such as subject learning.

**FIGURE 3:**
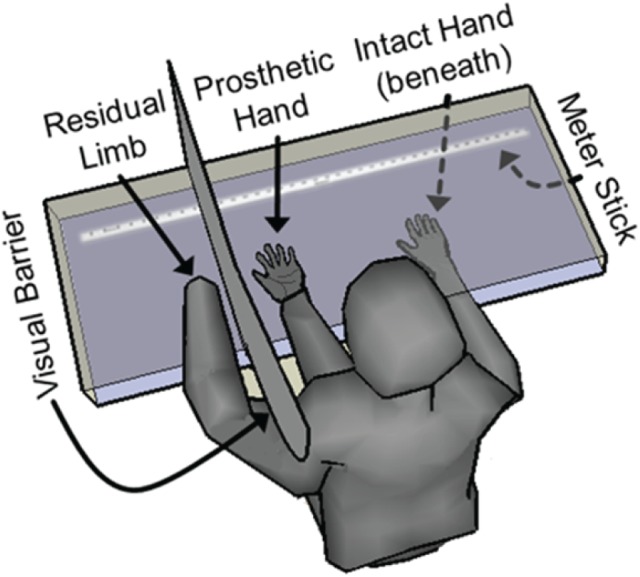
Embodiment quantification via measurement of shift in perceived hand location. The subject was seated facing a two-level plexiglass table. The subject’s residual limb was placed on the upper surface of the table and was shielded from his view with a visual barrier. The physical prosthetic hand was also placed on the upper surface in front of the subject along with a stuffed sleeve that was draped over the subject’s clothing to give the appearance of an arm extending from the subject’s left shoulder to the prosthetic hand. The subject’s right intact hand was placed on the lower surface, allowing it to pass beneath the prosthetic hand, the visual barrier and the residual limb. Both before and after each 4-min prosthetic-hand training period, the subject closed his eyes and moved his intact right hand laterally on the lower surface until he subjectively felt that his intact index finger was aligned with the index finger of his phantom hand. The perceived location of his phantom hand was documented using measurements from a meter stick. The shift in perceived phantom hand location during each trial was calculated as a metric of prosthesis embodiment.

Quantification of embodiment was performed by assessing a shift in perceived phantom hand position, as has been performed previously (Botvinick and Cohen, 1998; Collins et al., 2017). The physical prosthetic hand was placed palm up on a clear acrylic table, with the index-fingertip being positioned 13–19 cm to the right of the medial edge of the pronated residual left arm, which was also resting on the acrylic table (∼13 cm used in 8/10 experimental sets, ∼19 cm used in 2/10 experimental sets). A barrier was placed between the physical prosthesis and the residual limb so that the residual limb was not in sight. The subject donned a custom lab coat that was attached to the barrier. The coat included a conventional left sleeve for the subject’s residual left arm, plus an additional faux left sleeve that was stuffed and positioned in the subject’s view, projecting from his left shoulder to the wrist of the physical prosthesis, such that the prosthetic hand appeared to extend from this substitute left arm (**Figure 3**).

The intact right hand was placed on a lower acrylic surface, about 10 cm beneath the physical prosthesis and the residual limb, but was visible to the subject through the upper acrylic surface. The barrier between the physical prosthesis and the residual limb was not present on the lower acrylic surface, so that the intact right hand was free to pass beneath the physical prosthesis, the barrier, and the residual limb without impediment. The starting position of the intact right hand prior to a hand-movement saccade was fixed to be ∼49 cm to the right of the position of the prosthesis. A ruler was visible along the lower acrylic surface (but not touched by the subject), and a sliding T-square was placed on the ruler to allow for precise measurement of the subject’s intact index-finger location during the experiments.

Each embodiment experimental set consisted of four trials, one for each experimental condition. Each trial began by collecting a baseline assessment of the subject’s perceived phantom-hand location by placing his intact right hand at the designated starting position on the lower surface, closing his eyes, and moving his intact right hand along the lower acrylic surface until he felt that his right index-fingertip was aligned with his left phantom index-fingertip. The final position of his right-hand index finger was noted. A 4-min embodiment training period then began in which the subject was allowed to view the prosthesis during one of the following four experimental conditions: (1) motor control of the prosthesis; (2) sensory feedback from the prosthesis (experimenter pressed on the prosthesis sensor locations); (3) closed-loop control of the prosthesis (squeezing a ball or other object which allowed activation of the sensors); or (4) a control condition in which there was no motor control of or sensation from the prosthesis (visual fixation on the prosthesis). During the sensory feedback trials, the experimenter pressed only on the 3–4 active sensor locations in a random fashion, approximately once every 1–2 s. During the closed-loop control and motor control trials, the subject was able to move their phantom hand freely, although they often performed individuated movements (flexion or extension) of each DOF (thumb, index, middle, ring, pinky) on the prosthesis, cycling from one movement to the next every 1–3 s. During the visual fixation and sensory feedback trials, the subject relaxed their phantom hand in a resting position (indicated by low EMG and no motor-decode activity), although the subject was not explicitly instructed to keep his hand still. In order to create the most natural-feeling sensory experience, for both sensory feedback trials and closed-loop control trials, the USEA stimulation, and the subject’s resulting sensory experience, was dependent on the force applied to the sensors of the prosthetic hand. After the embodiment training period, the subject again placed his intact right hand at the start position on the lower surface, closed his eyes, and moved his right hand until he felt it was aligned with his phantom left hand. The difference between each pre-trial and post-trial perceived phantom hand position was used as an objective metric of embodiment. Trials were presented with a 4-min break between them which involved covering the physical prosthesis with a shroud and moving the residual limb and phantom hand as well as massaging, touching, and visualizing the residual limb to invoke disembodiment of the prosthetic hand.

**FIGURE 4:**
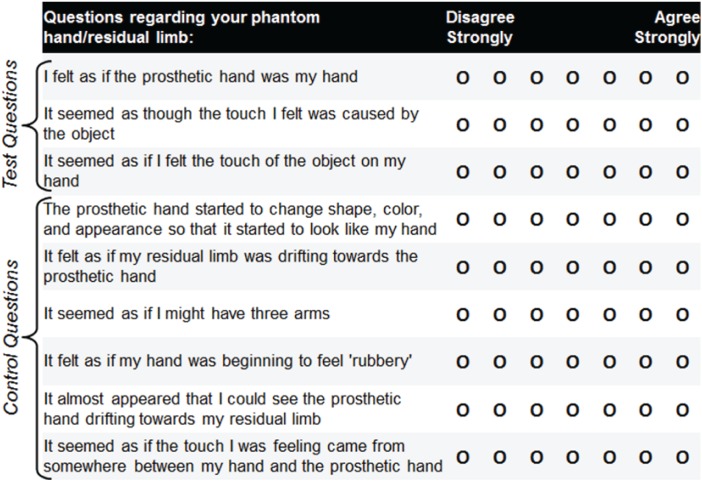
Embodiment survey questions modified from those used in other rubber-hand illusion tasks. The subject responded to nine survey questions following each prosthetic-hand training period. Three of the questions served as test questions to assess the level of prosthesis embodiment for the four different experimental conditions (closed-loop control, open-loop motor, open-loop sensation, visual fixation). The remaining six questions served as controls for task compliance and suggestibility. Eight different orderings of the survey questions were produced, and these different versions were delivered in block-random order. The subject’s overall impressions were also noted during the experiments.

Additionally, we collected subjective responses to survey questions related to embodiment of the limb after each trial. Survey questions were modified from those used in other rubber-hand illusion tasks (Botvinick and Cohen, 1998; Dummer et al., 2009; Rosén et al., 2009; Marasco et al., 2011; Schmalzl and Ehrsson, 2011; Walsh Lee et al., 2011; Kalckert and Ehrsson, 2012; Dobricki and de la Rosa, 2013; Caspar et al., 2015; Jenkinson and Preston, 2015; Collins et al., 2017), and included three predesignated test questions and six additional questions to control for task compliance and suggestibility (**Figure 4**). The subject indicated responses to the survey questions using a 7-point visual Likert scale. The nine different survey questions were arranged in different random orderings on eight different versions of the questionnaire, and the different versions were delivered in block-random order.

### Pain Evaluation

An extensive pre-implant pain evaluation was performed by a physician. A more concise method was used for routine pre-implant and post-implant evaluations, which consisted of asking the subject to rate his pain on a 0–10 scale, where a score of 10 was defined as the most intense pain he had ever experienced. This concise pain metric was used in place of more sophisticated pain surveys in order to save experimental time. Many different experiments were performed in post-implant sessions including USEA microstimulation, motor control, and closed-loop control of a virtual prosthesis in addition to embodiment experiments with a physical prosthesis. For two separate pre-implant sessions, and at the beginning and end of each post-implant experimental session, the subject’s pain was documented using the 0–10 rating scale. These questions were posed both for his chronic background phantom pain, which the subject described as being “always there,” and for phantom-pain episodes, which occurred periodically and were more intense. For periodic phantom-pain episodes, the duration, frequency, and intensity of episodes was also documented. The subject indicated that he had never had neuromas resected from his residual arm nerves.

Pain rating responses were analyzed for the following four conditions: all 74 experimental sessions, a subset of 55 experimental sessions involving closed-loop control, a subset of 8 experimental sessions involving only open-loop motor control, and a subset of 17 experimental sessions involving only open-loop stimulation. The subset of experiments involving open-loop motor control only consisted of seven full 3-h sessions in which no stimulation was provided and one 1-h session in which no stimulation was provided. The subset of experiments involving only open-loop stimulation involved one 3-h session in which the subject did not attempt any movements of his phantom hand, eleven 3-h sessions in which the subject only attempted to move his phantom hand for a total of 30 s, and one 2-h session in which the subject did not attempt any movements of his phantom hand, but which took place immediately following an open-loop motor session.

The subset of experimental sessions involving the embodiment experiments were all classified as closed-loop sessions because each embodiment session involved one experimental condition that had both motor control and sensory feedback. Direct comparisons between embodiment and phantom pain reduction were beyond the intended scope of the study.

### Statistical Analysis

Outliers in the survey question responses (more than 1.5 interquartile ranges above the upper quartile or below the lower quartile) were replaced by the next most extreme value (i.e., winsorizing) before statistical analyses (Tukey, 1977). If any test for normality (Anderson-Darling, Jarque-Bera and Lilliefors) indicated that the data were not normally distributed, then non-parametric tests were used. We found that the embodiment survey responses and pain score responses deviated from normality, whereas the proprioceptive shift did not.

For the proprioceptive drift, a statistical analysis of the perceived phantom hand location after the experimental condition relative to the perceived phantom hand location before the experimental condition was performed (paired, two-sided *t*-test) to evaluate the level of embodiment (positive shift toward prosthesis) for each of the four experimental conditions. For the embodiment survey responses, statistical analysis of the test question responses relative to the control question responses was performed (Mann–Whitney *U*-test) to evaluate the level of embodiment for each of the four experimental conditions. Lastly, for the pain score responses, statistical analysis of the pre-session and post-session pain rating responses was performed (paired, two-sided, Wilcoxon Sign Rank test) to evaluate the change in pain rating for the four conditions.

An omnibus parametric one-way analysis of variance (ANOVA) or non-parametric ANOVA (Kruskal–Wallis) was performed across all experimental conditions separately for the proprioceptive drift, embodiment survey and pain scores. When the omnibus ANOVA showed significance (i.e., for the proprioceptive drift and embodiment surveys), subsequent post-hoc multiple comparisons were performed using Fisher’s least significant difference procedure.

## Results

As evidenced by both proprioceptive drift and survey responses, the subject experienced embodiment of a physical prosthesis due to (1) open-loop visible motor control, (2) open-loop visible tactile feedback, and (3) closed-loop visible prosthesis control, but not after the visual-fixation condition. The subject also experienced a reduction in phantom pain across (1) all experimental sessions, (2) the subset of experimental sessions involving only open-loop motor control, (3) the subset of experimental sessions involving only open-loop stimulation, and (4) the subset of experimental sessions involving closed-loop control. Additionally, this reduction in phantom pain continued across the entire 14-month study.

### Embodiment: Shift in Perceived Hand Position

**FIGURE 5:**
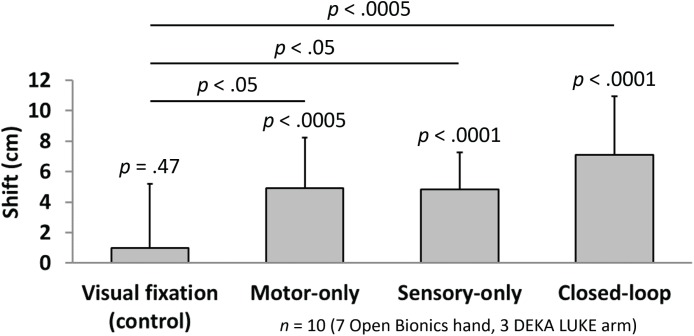
Quantification of perceived shift in limb position indicate the level of embodiment in the four experimental conditions (*n* = 10). Bars indicate the mean (± standard deviation) shift in perceived phantom limb position between the pre-trial and post-trial measurements for each of four experimental conditions. A significant shift toward the prosthesis (i.e., compared with no shift) was observed for each of the three test conditions (*t*-test; *p* < 0.0005 for motor-only, *p* < 0.0001 for sensory-only and closed-loop), whereas no significant shift toward the prosthesis was found for the visual fixation control condition (*t*-test; *p* = 0.47). In addition, each of the three test conditions (motor-only, sensory-only, and closed-loop) showed a greater level of embodiment compared with the visual fixation control condition (multiple comparisons; *p* < 0.05, *p* < 0.05, and *p* < 0.0005, respectively). No significant differences were found among the three test conditions.

Measures of proprioceptive shift showed selective embodiment in the three test conditions, relative to baseline and to the visual fixation control condition. The subject’s mean ± standard deviation perceived shifts in hand position toward the prosthesis were 0.99 ± 4.20 cm for visual fixation, 4.93 ± 3.31 cm for open-loop motor-only, 4.85 cm ± 2.42 cm for open-loop sensory stimulation, and 7.09 ± 3.86 cm for closed-loop control (**Figure 5**). A statistically-significant shift in the perceived hand position toward the prosthesis was observed for open-loop motor control (*p* < 0.0005), open-loop sensory feedback (*p* < 0.0001) and closed-loop control (*p* < 0.0001). Importantly, there was no evidence of a significant shift for the visual fixation condition (*p* = 0.47). In addition, there was a statistically-significantly difference in proprioceptive shift among the four experimental conditions (ANOVA, *p* < 0.005). Further, each of the three test conditions (open-loop motor control, open-loop sensory feedback and closed-loop control) showed a greater level of embodiment compared to the visual fixation control condition (*p* < 0.05, *p* < 0.05, and *p* < 0.0005, respectively).

No significant differences were found among the three test conditions (open-loop motor vs. open-loop sensory, *p* = 0.96; open-loop motor vs. closed-loop, *p* = 0.18; open-loop sensory vs. closed-loop, *p* = 0.16). Although it was not significant, the closed-loop condition showed a slight trend toward increased proprioceptive shift relative to the open-loop motor and open-loop sensory conditions.

### Embodiment: Survey Results

**FIGURE 6:**
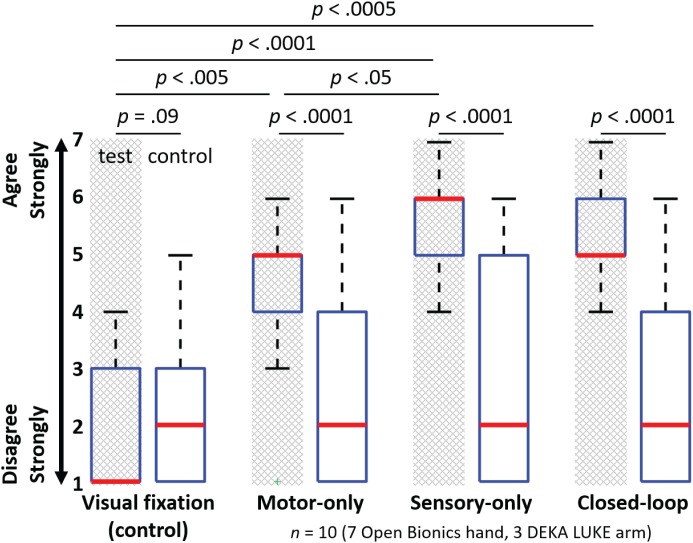
Survey question responses indicate the level of embodiment in the four experimental conditions (*n* = 10). Figure shows the median (bold red line), interquartile range (blue box), 1.5 times the interquartile range (black whiskers) and one outlier (green +). The motor-only, sensory-only, and closed-loop test conditions each exhibited a significantly higher response on the test questions compared with the control questions (*U*-test; *p* < 0.0001 for each of these three conditions), whereas no such difference was found for the visual fixation control condition (*U*-test; *p* = 0.09). Each of the three test conditions (open-loop motor control, open-loop sensory feedback and closed-loop control) showed a greater level of embodiment compared with the visual fixation control condition (multiple comparisons; *p* < 0.005, *p* < 0.0001, and *p* < 0.0005, respectively). The open-loop sensory feedback condition also showed a significantly higher level of embodiment relative to the open-loop motor control condition (multiple comparisons; *p* < 0.05).

Test survey questions also demonstrated selective embodiment in the three test conditions, relative to the control questions and to the visual fixation condition. The subject’s median (and IQR) response to the test survey questions was 1 (2) for visual fixation, 5 (1) for open-loop motor control, 6 (1) for open-loop sensory feedback, and 5 (1) for closed-loop control (**Figure 6**). We compared the pooled Likert ratings from the three test questions to the pooled Likert ratings from the six control questions for each of the four experimental conditions. Open-loop motor, open-loop sensory, and closed-loop test conditions each exhibited a significantly higher response on the test questions compared with the control questions (*p* < 0.0001 for each of these three conditions), whereas no significant difference was found for the visual fixation control condition (*p* = 0.09). In addition, there was a statistically significant difference in the Likert ratings for the test questions among the experimental conditions (Kruskal–Wallis, *p* < 0.0001). Each of the three test conditions (open-loop motor, open-loop sensory, and closed-loop control) showed a greater level of embodiment compared with the visual fixation control condition (*p* < 0.005, *p* < 0.0001, and *p* < 0.0005, respectively).

In addition, the open-loop sensory condition showed a significantly higher level of embodiment relative to the open-loop motor condition (*p* < 0.05). No significant differences were found among the remaining conditions (open-loop motor vs. closed-loop, *p* = 0.42; open-loop sensory vs. closed-loop, *p* = 0.08). Although it was not significant, the sensory-only condition showed a trend toward increased embodiment relative to the closed-loop condition. The increased embodiment associated with the open-loop sensory condition may be attributed to the fact that some of the test survey questions had a sensory component (**Figure 4**) and that sensory percepts may have been more readily activated by the experimenter than by closed-loop object manipulation.

The subject’s informal comments were also helpful for assessing embodiment. After an open-loop sensory embodiment trial, the subject stated, “It does make a difference on the [stimulation]. It really feels like you’re squeezing my thumb, ’cause where you’re squeezing is where the stimulation is.” Following a closed-loop embodiment trial, the subject stated, “I want to clasp my hands together,” at which point he massaged, touched, and squeezed the prosthetic hand with his intact hand during closed-loop control for about 20 s. His use of the wording “my hands” is consistent with a subjective sense of embodiment.

The subject also indicated that although his perceived range-of-motion of movement control of the digits of his phantom hand was normally quite limited, active movement of the digits of the physical prosthetic hand with visual feedback seemed to open his phantom hand. At about 10 weeks post-implant (with experimental sessions several times per week), he reported that the range-of-motion of his phantom digits was beginning to widen at times, allowing him to open and close some digits of his phantom hand, even outside of the experimental sessions.

### Phantom Pain Reduction

The subject described two distinct types of phantom pain: (1) consistent background phantom pain, described as sharp and burning; and (2) sporadic intense phantom pain events that typically lasted several seconds, but that occurred only 1–4 times per day. Sporadic phantom pain episodes rarely occurred during experimental sessions, so the effect of the experiments on this type of pain was not quantified. The subject’s background phantom pain increased to a level of 6 during the first 10 days after the implant and then settled to a relatively stable median (and IQR) pain score of 4 (1). The maximum subjective pain score ever reported by the subject was a 7, which occurred while the subject was at home between sessions. The subject’s median pre-implant phantom pain was a 4.25 (0.5).

**FIGURE 7:**
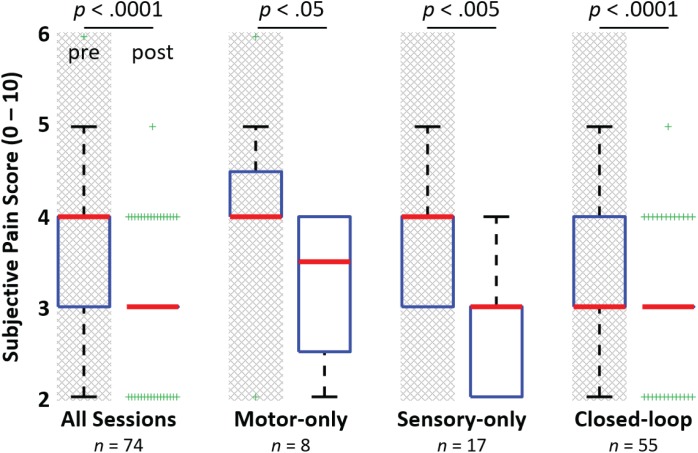
Reduction in phantom limb pain after experimental sessions. The figure shows the median (bold red line), interquartile range (blue box), 1.5 times the interquartile range (black whiskers) and the outliers (green +’s). A significant reduction in phantom limb pain (Rank test; *p <* 0.0001) was observed between the subject’s pre-session and post-session subjective pain ratings for the 74 experimental sessions leading up to 14 months post-implant. A significant reduction in phantom pain was also observed for the subsets of sessions involving only motor control of a virtual or physical prosthesis, open-loop nerve microstimulation via USEAs in sensory-only sessions, and closed-loop sessions involving motor control and sensory feedback (Rank test; *p* < 0.05, *p* < 0.005, and *p* = 0.0001, respectively). Although full pain relief was not provided for any of these cases (e.g., overall, a median of 25% pain reduction was observed), the subject indicated that pain reduction is important and helpful for continuing with activities of daily living.

**FIGURE 8:**
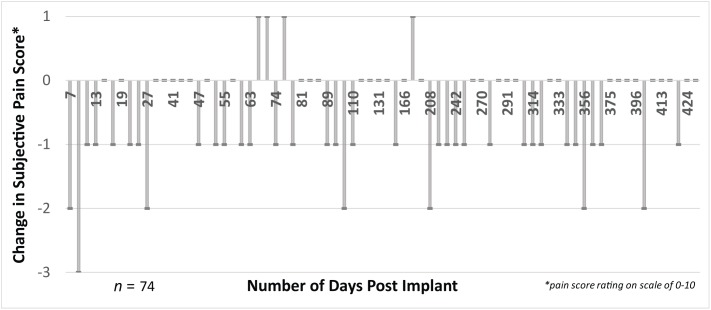
There was a consistent decrease (difference in post-session vs. pre-session) in pain scores measured in 74 experimental sessions. We collected subjective ratings of phantom pain across time up to 14 months post-implant for both pre-session phantom pain and post-session phantom pain. Most experimental sessions resulted in a reduction in phantom pain, evidenced by a negative difference between the post-session and pre-session pain score. This reduction was relatively consistent across the entire 14-month duration of the study. The subject continued his use of prescription medications for treatment of phantom pain during the duration of the implant (e.g., gabapentin and tramadol).

The subject’s verbal scoring of his background phantom pain indicates a statistically significant reduction in phantom pain (after vs. before session) across all 74 experimental sessions (*p* < 0.0001, **Figure 7**). This reduction in phantom pain was consistent across the entire 14-month duration of the study (**Figure 8**). The median pre-session pain score was 4 (1), and the median post-session pain score was 3 (0), yielding a 25% median reduction in phantom pain. A statistically significant reduction in phantom pain was also present for the subset of experimental sessions that involved only open-loop motor control (*p* < 0.05), the subset of experimental sessions that involved only open-loop stimulation (*p* < 0.005), and the subset of experimental sessions that involved closed-loop control (*p* < 0.0001). There was not a statistically significantly difference in the change in pain rating response among these three experimental conditions (Kruskal–Wallis, *p* = 0.097).

## Discussion

We used USEAs implanted in residual peripheral arm nerves and iEMGs implanted in residual limb muscles to provide one human subject with touch sensation, motor control, and ultimately closed-loop control of physical and virtual prosthetic hands. The subject embodied the physical prosthetic hands in cases of open-loop motor control, open-loop sensory feedback, and closed-loop motor control with sensory feedback, and the level of prosthesis embodiment was significantly increased compared with embodiment after a visual fixation condition (e.g., similar to a cosmetic prosthesis). Embodiment experiments were not performed with the virtual hand. The subject also reported a reduction in phantom pain during experimental manipulations that included nerve microstimulation, motor control of a virtual prosthesis, closed-loop control of a virtual prosthesis, and sensory, motor, and closed-loop interaction with a physical prosthesis.

This work further demonstrates that both open-loop sensory and open-loop motor conditions can cause prosthesis embodiment. Additionally, this work shows that open-loop sensory (USEA nerve microstimulation alone) and open-loop motor control of a prosthesis were independently able to cause a reduction in subject reported phantom pain. Importantly, for both prosthesis embodiment and phantom pain reduction, the closed-loop condition did not yield significant improvements over the open-loop conditions.

### Embodiment Results

Embodiment metrics included the objective indication of the subject’s perceived location of his phantom hand before and after an embodiment training period, as well as subjective responses to survey responses. Previous studies have used perceived hand location extensively as an embodiment metric (Botvinick and Cohen, 1998; Tsakiris et al., 2006; Rosén et al., 2009; Kalckert and Ehrsson, 2012; Caspar et al., 2015; Jenkinson and Preston, 2015; Romano et al., 2015; Fuchs et al., 2016; Collins et al., 2017). However, the present report represents the first use of the shift in perceived phantom hand location as a prosthesis embodiment metric for closed-loop controlled prostheses where feedback is provided via peripheral nerve microstimulation. We found this metric to be both reliable and repeatable in providing an objective measurement of prosthesis embodiment under open-loop motor control, open-loop sensory feedback, and closed-loop motor control with sensory feedback.

Several studies have demonstrated the ability to induce embodiment of a rubber or prosthetic hand through sensory feedback alone in intact individuals and amputees. More recently studies have now also shown that movement, and the natural proprioception associated with it, is enough to induce a sense of embodiment over a motorized hand (Tsakiris et al., 2006; Dummer et al., 2009; Rosén et al., 2009; Walsh Lee et al., 2011; Kalckert and Ehrsson, 2012; Caspar et al., 2015; Jenkinson and Preston, 2015). Our results confirm these findings and expand upon them by providing direct comparisons among the visual fixation (control), motor-only, sensory-only and closed-loop conditions.

The subject’s shift in perceived phantom hand location suggests that the strength of the embodiment illusion was roughly comparable across test conditions. Each one of the test conditions produced a significant level of embodiment relative to the control condition, but none of the test conditions were significantly different from one another. Although the closed-loop condition resulted in the largest proprioceptive shift among the test conditions, as might have been expected given that both the sensory-only and motor-only conditions produced proprioceptive drift individually, the trend was not significant.

Speculatively, several factors may have diminished or obscured potentially significant differences among the three test conditions. For one, differences among the test conditions may be modest, and hence datasets might require greater sample sizes in order to reveal statistical significance. In addition, using the prosthesis for a longer duration may have also been necessary to produce greater amounts of proprioceptive shift and hence potentially larger differences among the three test conditions. Lastly, inconsistent sensor activation and the resulting sensory feedback in the closed-loop condition may have limited embodiment under the closed-loop condition. Alternatively, a ceiling effect may have been present and sensorimotor interactions may not be necessary to produce maximal embodiment as quantified by proprioceptive drift and survey questions.

The subject’s response to the embodiment survey questions also demonstrated a significant level of embodiment for each test condition relative to the control condition. However, this metric of embodiment was different from proprioceptive drift in that the sensory-only condition caused a significantly greater level of embodiment compared with the motor-only condition and had a strong, but non-significant trend toward greater embodiment than the closed-loop condition. The increased embodiment associated with the sensory-only condition may be attributed to the fact that some of the test survey questions had a sensory component (**Figure 4**). Embodiment results can differ based on the question being analyzed (Dummer et al., 2009). In addition, the sensory percepts may have been more readily activated by the experimenter’s precise activation than by the subject’s closed-loop object manipulation.

### Embodiment Limitations and Extensions

It is believed that illusion of ownership or embodiment of an artificial body or body part, while malleable, depends on congruence (plausible anatomical orientation), temporal and spatial synchrony (between visual and proprioceptive or tactile feedback), and “bodily resemblance” (Schmalzl and Ehrsson, 2011). To this end, we anticipate that the embodiment effect with amputees controlling advanced prostheses will be strongly dependent on the extent, naturalism, spatial accuracy, and latency of the restored sensation and motor control. The embodiment levels demonstrated here may be reduced due in part to limits in controllable movement and sensory feedback.

One important limitation in the motor-only and closed-loop condition may be the inaccuracies in the prosthetic control algorithm. All the studies exploring the embodiment associated with a moving hand either use the hand kinematics of an intact individual to control the motorized hand (Tsakiris et al., 2006; Dummer et al., 2009; Walsh Lee et al., 2011; Kalckert and Ehrsson, 2012; Caspar et al., 2015; Jenkinson and Preston, 2015) or EMG signals from the residual limb of an amputee (Rosén et al., 2009; Schiefer et al., 2016). Although the relative amount of temporal synchrony between these two conditions can be determined, the spatial synchrony and congruence cannot be compared, as it is difficult to quantify the exact relationship between the amputee’s intended movement and the algorithm’s output movement. Future studies, comparing the level of embodiment associated with movement predicted by direct hand kinematics relative to residual-forearm EMG-based estimations of hand kinematics may help elucidate what enhancements in embodiment may arise from improvements in prosthetic control algorithms.

The sensory feedback used for these embodiment experiments was limited to three or four cutaneous sensory percepts. These percepts were evoked via single-electrode or multi-electrode stimulation through four different subsets of USEA electrodes tied to individual prosthesis sensors. Future experiments should use the rich selection of sensory feedback that can be provided by USEAs to provide extensive sensory feedback via many sensors. Additionally, more-biomimetic stimulation patterns using multielectrode, mixed-receptor-type stimulation tied to each sensor may evoke more naturalistic sensations and improved embodiment and/or phantom pain relief (Saal and Bensmaia, 2014). Self-touching of prosthesis sensors may also assist in generating a stronger sense of embodiment via restored tactile feedback. It is also important to note that embodiment conditions explored in this study lacked extrinsically provided proprioceptive feedback, which has been shown to contribute to embodiment (Walsh Lee et al., 2011). Endogenous proprioceptive feedback from residual extrinsic hand muscles and efference copy may have remained.

We hypothesize that the level of prosthesis embodiment will increase with more sophisticated motor control and sensory feedback and ultimately, more extended use in ADL. Future work should include a quantification of the level of embodiment of the prosthetic limb as a function of: (1) the number of sensors used for sensory feedback; (2) the range of sensation intensity encoded by prosthesis sensors; (3) the number of DOF included in the motor decode; (4) the precision of proportional motor control; and 5) the extent and duration of use. Fitt’s law is a functional performance metric that indicates that the time required to complete a functional motor task is proportional to the task’s complexity (Fitts, 1954). We speculatively propose that a parallel law exists for psychological or emotional impact metrics, such as embodiment or phantom pain relief, in which the level of embodiment or phantom pain reduction may increase in proportion to the extent of naturalistic sensory feedback and/or motor control provided.

We also anticipate that the nature of the neural interface used for restoration of sensation will influence the extent of prosthesis embodiment by indirectly determining the capabilities for sensory encoding. In informal pre-implant testing using intact hands, we subjectively observed that the rubber hand illusion was more salient when multiple different hand locations were touched in a seemingly unpredictable pattern. During prosthesis embodiment trials, our subject indicated verbally that touch of the prosthesis palm and thumb were particularly meaningful to him and seemed to enhance the sense of embodiment. In future studies, more sensors should be integrated into the prosthetic hand and coupled to additional electrodes for restoring sensory percepts representing, for example, the tip of each digit, the midsection of each digit, multiple areas of the palm, the lateral edge of the hand, and the back of the hand. The scotoma effect, or the tendency for sensory perception to “fill in” between adjacent sites of sensation, may enable perception of full-hand cutaneous sensation even in locations where tactile sensors are not present.

The metrics used in this manuscript are limited in their ability to accurately quantify embodiment. Responses to survey questions can vary on the basis of the question being analyzed (Dummer et al., 2009), and proprioceptive drift may be dissociated from the feeling of embodiment (Rohde et al., 2011). Furthermore, both metrics may have varied effectiveness among individuals (Bekrater-Bodmann et al., 2012).

Despite these limitations, the ability for the test conditions to elicit a sense of embodiment relative to the control condition is a testament to the synchrony and congruence of the motor control and sensory feedback algorithms, as it has been shown that inconsistencies or delays between the intended movement and actual movement can eliminate the sense of embodiment (Caspar et al., 2015). With improved sensory feedback and motor control, we hypothesize that the closed-loop condition will ultimately provide increased embodiment for both proprioceptive drift and questionnaires.

### Phantom Pain Discussion and Extensions

Phantom pain reduction was reported by our subject for many experimental sessions, which included USEA nerve microstimulation, motor control, and closed-loop control of a virtual or physical prosthesis. When we first questioned him about his sensory awareness of his phantom hand, the subject indicated, “Probably the reason that I can feel it’s there is the phantom pain.” He reported that he had previously attempted mirror-box therapy (Ramachandran and Rogers-Ramachandran, 1996), transcutaneous electrical nerve stimulation therapy, and magnet therapy for phantom pain relief with no perceived improvement. During his first experimental session, while he was controlling the movements of the virtual hand, he indicated, “That just feels good, actually—seeing it open all the way up.” He later stated, “It’s interesting, ‘cause the mirror [box] didn’t give me that same sensation.”

Although we observed only modest pain relief due to experimental manipulations, the subject indicated that phantom pain reduction is important, helping to keep the pain at a manageable level. For example, the subject stated that although his pain medications do not completely relieve him of his phantom pain, they keep it at a level which is bearable and which allows him to carry on with ADL. Even the modest reductions on the scale noted herein may have additional benefits. Future studies should also make use of more sophisticated pain questionnaires that are validated for the amputee population in order to better estimate the clinical relevance of the pain reduction detailed here. Further, daily use of a dexterous, sensorized prosthesis may provide greater reductions in phantom pain and extended prosthesis embodiment. Future studies investigating long-term phantom pain reduction and its relationship to prosthesis embodiment may help elucidate the underlying mechanism of phantom pain.

The mechanisms of phantom pain formulation are not well understood, with evidence suggesting peripheral and/or central mechanisms (Subedi and Grossberg, 2011; Vaso et al., 2014; Yanagisawa et al., 2016). Although we did not formally assess the specific nature of the phantom pain reduction, the location of the subject’s phantom pain reduction seemed at times to be related to the innervation distribution of the nerve being stimulated. For example, median-nerve stimulation sessions often resulted in pain reduction on the first, second, and third digits, but not on the fourth and fifth digits. In addition, when describing the pain reduction, the subject often stated the pain reduction occurred in the same location as the perceived stimulation and that the modality of pain shifted from a “constant, background burning” to a “pressure-like” sensation resembling that of the stimulation.

Visual-motor integration coupled with internal efference copy, such as is generated during dexterous prosthesis motor control, represents the convergence of many rich correlative signals that seem capable of masking perception of background phantom pain. We anticipate that advanced closed-loop control of a sophisticated prosthesis that is attached to the limb and used for daily tasks may represent an even stronger masking signal, potentially providing more substantial pain reduction.

## Conclusion

The challenges associated with limb loss include not only functional deficits, but also the emotional difficulty associated with losing a body part, and in many cases chronic phantom pain. These psychological and emotional factors may be more important to patients’ overall health and well-being than functional outcomes (Dolan, 2002; Pressman and Cohen, 2005; Zhang and Li, 2005). The results presented here extend previous studies by showing that USEA stimulation and iEMG- and neural-based movement decodes can provide meaningful psychological benefits to amputees. The subject embodied prosthetic hands, as evidenced by a shift in his perceived phantom hand location toward the prosthesis and by his response to survey questions. Additionally, the subject consistently reported a reduction in phantom pain after movement decode and microstimulation sessions. This work represents the first long-term and systematic report of prosthesis embodiment and pain reduction during closed-loop prosthesis use in a human amputee.

Restoration of sophisticated prosthesis motor control and prosthesis sensation provided a sense of limb-restoration that was meaningful to our subject, and which may assist future amputees improve their emotional health. Ultimately, we envision development of a take-home, wearable, closed-loop prosthesis system that may serve not only as a helpful tool, but also as a limb replacement that provides subjective psychological as well as physical benefits.

## Data Availability Statement

The datasets for this manuscript are not publicly available due to HIPAA compliance requirements. Due to the limited number of subjects involved in this study, the University of Utah Institutional Review Board has ruled that the data cannot be truly de-identified. Requests to access the datasets should be directed to Gregory A. Clark, Greg.Clark@utah.edu.

## Ethics Statement

This study was carried out in accordance with the recommendations of the University of Utah Institutional Review Board, and the Department of the Navy Human Research Protection Program, with written informed consent from all subjects. All subjects gave written informed consent in accordance with the Declaration of Helsinki. The protocol was approved by the University of Utah Institutional Review Board and representatives of the Department of the Navy Human Research Protection Program.

## Author Contributions

DP designed and planned experiments, collected embodiment data for the Open Bionics hand, collected pain scores, instrumented the Open Bionics hand with sensors, developed software and hardware for closed-loop sensory feedback via hand sensors, performed data analysis, and drafted the manuscript. JG collected embodiment data for the DEKA LUKE Arm, collected pain scores, developed software for closed-loop sensory feedback for the DEKA LUKE Arm, and assisted with drafting of the manuscript. DK developed the Open Bionics hand’s on-board software and integration with sensory hardware, assisted with software development for sensory feedback, motor control, and closed-loop control, performed pre-surgery implant hardware verification and sterile packaging, assisted with implant and explant surgeries, and performed data collection. CD printed and assembled the Open Bionics hand, provided clinical support, developed methods for collecting pain scores, and assisted with data analysis. SW developed motor control and closed-loop control software and hardware, and assisted with experiments and collection of data. TD developed motor control, closed-loop, and sensory stimulation software and hardware and provided extensive engineering and clinical contribution to the development of the experiments and devices, and helped with data collection. DH implanted and explanted the USEAs and iEMGs, provided clinical oversight throughout the study, and oversaw development of experiments and protocols. GC oversaw and led development of all methods, experiments, and protocols, and assisted with drafting of the manuscript. All the authors contributed to the revision of the manuscript.

## Conflict of Interest Statement

Author JG declares that the research was conducted in the absence of any commercial or financial relationships that could be construed as a potential conflict of interest. Authors DP, DT, CD, SW, TD, DH, and GC declare the following patents as conflicts of interest: Microelectrode Array System with Integrated Reference Microelectrodes to Reduce Detected Electrical Noise and Improve Selectivity of Activation. **Patent number:** 8359083. **Type:** Grant. **Filed:** April 2, 2009. **Issued:** January 22, 2013. **Assignee:** University of Utah Research Foundation. **Inventors:** Gregory A. Clark, David James Warren, Noah M. Ledbetter, Marcy Lloyd, Richard A. Normann. System and Method for Electrically Shielding a Microelectrode Array in a Physiological Pathway from Electrical Noise. **Patent Number:** 8639312. **Type:** Grant. **Filed:** December 10, 2009. **Issued:** January 28, 2014. **Assignee:** University of Utah Research Foundation. **Inventors:** Gregory Arthur Clark, David J. Warren, Noah M. Ledbetter. Signal processing for decoding intended movements from electromyographic signals **Application number:** WO2018026842A1. **Status:** Filed Internationally. **Type:** International Application. **File date:** July 28, 2017. **Issued:** February 8, 2018. **Assignee:** The University of Utah Research Foundation. **Inventors:** Suzanne M. Wendelken, Tyler Davis, David T. Kluger, Christopher Duncan, David J. Warren, David M. Page, Gregory A. Clark. A medical device implant carrier for fragile medical implants **Application number:** WO2018023026A1. **Status:** Filed Internationally. **Type:** International Application. **File date:** July 28, 2017. **Issued:** February 1, 2018. **Assignee:** The University of Utah Research Foundation. **Inventors:** David T. Kluger, Gregory A. Clark, Douglas T. Hutchinson, David M. Page, Suzanne M. Wendelken.
